# Surgical Treatment for Profunda Femoris Artery Aneurysms: Five Case Reports

**DOI:** 10.1155/2015/375278

**Published:** 2015-04-30

**Authors:** Kimihiro Igari, Toshifumi Kudo, Takahiro Toyofuku, Yoshinori Inoue

**Affiliations:** Division of Vascular and Endovascular Surgery, Department of Surgery, Tokyo Medical and Dental University, 1-5-45 Yushima, Bunkyo-ku, Tokyo 113-8519, Japan

## Abstract

Profunda femoris artery aneurysm (PFAA) is an extremely rare entity, with most cases being asymptomatic, which makes obtaining an early diagnosis difficult. We herein report a case series of PFAA, in which more than half of the PFAAs, which presented with no clinical symptoms, were discovered incidentally. All PFAAs were treated surgically with aneurysmectomy with or without vascular reconstruction. In cases involving a patent superficial femoral artery (SFA), graft replacement of the profunda femoris artery (PFA) is not mandatory; however, preserving the blood flow of the PFA is necessary to maintain lower extremity perfusion in patients with occlusion of the SFA. Therefore, the treatment of PFAAs should include appropriate management of both the aneurysmectomy and graft replacement, if possible.

## 1. Introduction

Profunda femoris artery aneurysm (PFAA) is an uncommon condition, accounting for only 0.5% of peripheral aneurysms and only 1–2.6% of all femoral artery aneurysms [1]. Most PFAAs are pseudoaneurysms resulting from iatrogenic injury or trauma [2], while true aneurysms of the profunda femoris artery (PFA) are much less frequent. Aneurysmal changes in PFA have been reported to be rare because several muscles cover the PFA in this anatomical location [3]. Based on the anatomical location, diagnosing small and asymptomatic PFAAs is difficult. PFAAs may cause symptoms of local venous and nerve compression, which may lead to distal venous congestion and local pain. Furthermore, these aneurysms are occasionally complicated with distal embolism, limb-threatening ischemia, and rupture [4]. We herein report the results of our experience with surgical treatment for true PFAAs.

## 2. Case Presentation

### 2.1. Patients and Methods

A retrospective review was performed on all patients with a diagnosis of PFAA who underwent surgical treatment at Tokyo Medical and Dental University Hospital between January 2005 and December 2014. All subjects provided their informed consent, and approval was obtained from our Institutional Review Board for a retrospective review of the patients' medical records and images. The inclusion criterion was aneurysmal dilatation of a PFA of more than 20 mm, based on preoperative imaging findings. Cases of pseudoaneurysms of PFAA due to trauma were excluded, and only true aneurysms were included. The medical records were abstracted to include basic demographic information, preoperative symptoms, aneurysm size measurements, intraoperative findings, perioperative complications, and long-term imaging findings. The characteristic features of the patients are given in Table 1.

### 2.2. Case 1

A 76-year-old asymptomatic male presented for follow-up magnetic resonance imaging (MRI) after open surgical repair of an abdominal aortic aneurysm (AAA). MRI showed a PFAA measuring 45 × 40 mm on the right side of the thigh. The aneurysm was successfully resected under general anesthesia without vascular reconstruction, as the superficial femoral artery (SFA) was patent, and the distal portion of the PFA was very small, making it unsuitable for revascularization. The patient's postoperative course was uneventful, and the postoperative ankle brachial pressure was within the normal limits without any lower limb ischemia.

### 2.3. Case 2

**Figure 1:**
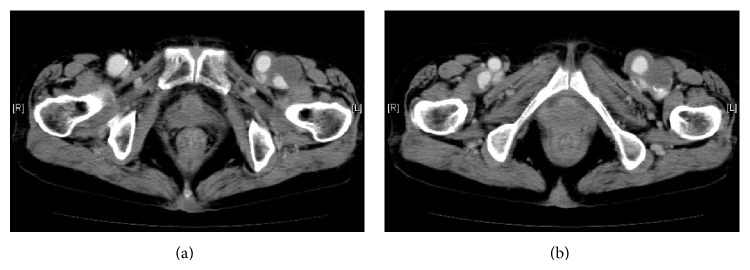
Computed tomography showed bilateral common femoral artery aneurysms (a) and bilateral profunda femoris artery aneurysms (b).

A 69-year-old female presented with pain and swelling of the left thigh. Computed tomography (CT) showed a left PFAA measuring 34 × 24 mm. Furthermore, CT detected a right PFAA measuring 25 × 22 mm, without clinical symptoms, and the bilateral common femoral arteries (CFAs) showed aneurysmal changes (Figure 1). The bilateral CFA aneurysms (CFAAs) and PFAAs were resected under general anesthesia, and resected bilateral CFAAs were interposed using the prosthesis measuring 8 mm in size. Bilaterally, bypass grafting was performed from the interposed prosthesis which measured 8 mm in size to the distal part of PFA by a vascular prosthesis measuring 6 mm in size. The patient's postoperative course was uneventful, without any evidence of lower limb ischemia.

### 2.4. Case 3

**Figure 2:**
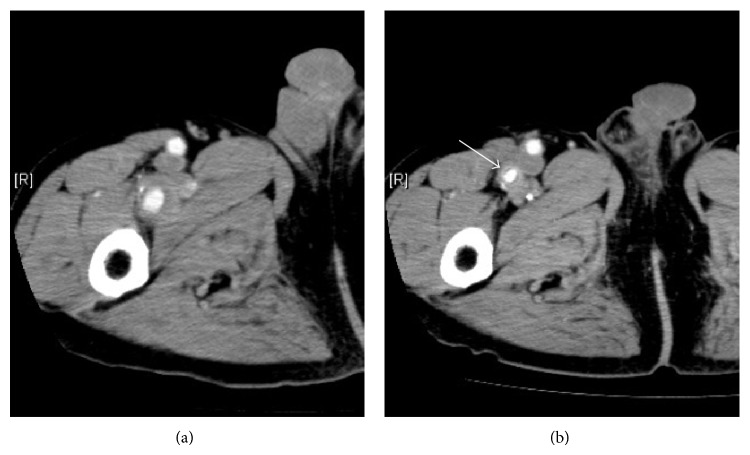
(a) Preoperative computed tomography exhibited a 22 mm right profunda femoris artery aneurysm with an intraluminal thrombus. (b) Postoperative computed tomography revealed a patent replaced prosthetic graft (white arrow).

A 73-year-old asymptomatic male presented for follow-up CT after open surgical repair of AAA and bilateral common iliac artery aneurysms and an assessment of an untreated thoracic artery aneurysm measuring 40 mm in size. CT exhibited a PFAA measuring 25 × 22 mm on the right side of the thigh (Figure 2(a)). The aneurysm was successfully resected under general anesthesia with revascularization from the proximal to the distal part of the PFA using an 8 mm prosthesis (Figure 2(b)). The patient developed a wound infection after the operation; however, it healed with conservative treatment.

### 2.5. Case 4

**Figure 3:**
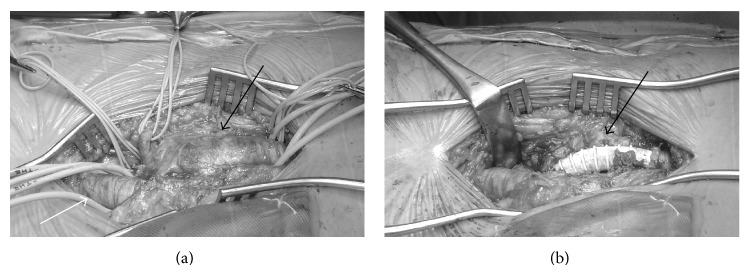
(a) The intraoperative findings showed the controlled profunda femoris artery (black arrow) and superficial femoral artery (white arrow), and (b) the aneurysmectomy was performed with graft interposition (black arrow). The patient's head was to the right.

A 65-year-old asymptomatic female presented for the ultrasonography to evaluate the varicose vein. US showed the 25 mm sized mass on her right groin. Further contrast enhanced CT scanning showed the right PFAA measuring 26 × 25 mm. Under general anesthesia, the SFA and the proximal and distal part of PFAA were well controlled; the aneurysmectomy was successfully performed with the interposed 8 mm prosthetic graft placed between the proximal and distal PFA (Figure 3). Postoperative course was uneventful, without lower limb ischemia.

### 2.6. Case 5

**Figure 4:**
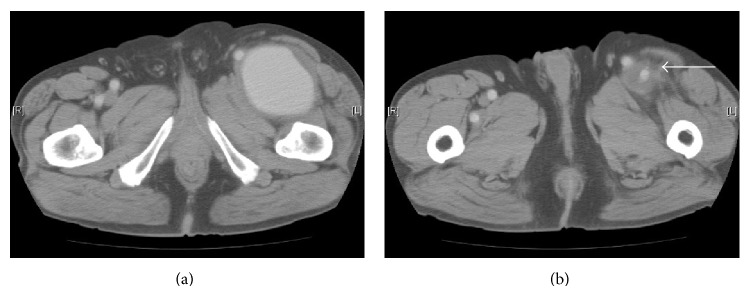
Computed tomography showed a 78 × 86 mm left profunda femoris artery (PFA) (a), which extended to the distal part of the left PFA (white arrow) (b).

A 70-year-old male presented with a palpable mass and pain in the left thigh. Contrast enhanced CT revealed a left PFAA measuring 86 × 76 mm (Figure 4). The aneurysm was successfully resected under general anesthesia without revascularization, as the distal portion of the PFA was very small, meaning that it was too difficult to revascularize, and the SFA was patent. The patient's postoperative course was uneventful, without any evidence of lower limb ischemia.

### 2.7. Surgical Procedures and Postoperative Results (Tables 2 and 3)

A total of six PFAAs were resected in five patients. The mean operative time was 130 minutes (range: 81–210 minutes) and the mean amount of intraoperative blood loss was 122 mL (range: 15–594 mL); therefore, none of the patients required a blood transfusion. Four of the six PFAAs were interposed with a prosthetic graft, and, in case 2, the bilateral PFAAs and CFAAs were resected simultaneously with revascularization. Two of the six PFAAs were treated with ligation without revascularization because the distal part of each PFAA was located too far to achieve revascularization. The pathological findings of the resected aneurysms showed degenerative and atherosclerotic changes in all six PFAAs.

None of the patients exhibited lower limb ischemia after the surgical procedures and all were discharged successfully. During the long-term follow-up period (median: 18 months, range: 8–76 months), no patients presented with signs of lower limb ischemia, and all of the interposed grafts remained patent.

## 3. Discussion

Previous reviews of published cases have indicated that patients with PFAA often have synchronous aneurysms, occurring in 65–75% of cases, including AAA and popliteal artery aneurysms [5]. Bilateral PFAAs occur in only 5% of patients with PFAAs, in contrast to femoral artery aneurysms, which occur bilaterally in the majority of cases [2]. In our case series, three of five patients with PFAAs had other synchronous aneurysms (60%), and bilateral PFAAs were noted in one case (20%); these findings are compatible with those of previous reviews. Cutler and Darling classified femoral artery aneurysms according to the relationship between the CFA and CFA bifurcation. Type I involves aneurysmal changes localized in the CFA, whereas, in type II, the aneurysmal changes extend to the proximal part of the superficial femoral artery (SFA) and PFA [6]. According to this classification, the current case 2 can be classified as type II.

It has been reported that PFAAs are much more common in males (92–100%) than in females, and most PFAAs are discovered in the sixth to seventh decades of life [3]. Furthermore, it has been reported that most patients with PFAAs have a decades-long history of smoking and hypertension [7]. In the current study, the details of our cases are comparable to those of previous reports concerning epidemiological findings, in particular, that all of the patients had a smoking habit, which may exhibit a significant correlation with the onset of PFAA.

Although patients with PFAAs usually remain asymptomatic and the lesions are discovered incidentally, such patients may present with symptoms related to local compression, thrombosis, or embolism, with consequent rupture. Compression-related symptoms include groin swelling, pain, and pulsatile masses [2]. In our cases, four of the six PFAAs were asymptomatic and found incidentally, and the other two presented with local compressive symptoms. However, PFAAs may present with acute ischemic symptoms due to thrombosis and/or embolism of distal vessels [8]. Furthermore, rupture is believed to be a more common presentation for PFAAs than other peripheral aneurysms [1] and may carry a high risk of limb loss and even mortality. Therefore, early diagnosis and treatment are essential in such cases.

Following the diagnosis of PFAA, elective surgical repair is recommended whenever the patient's general condition allows for surgical intervention [3]. A reasonable recommendation is to repair PFAAs measuring over 20 mm in diameter [1]. However, a recent study reported that acute complications are rare in cases of femoral artery aneurysms < 35 mm in diameter and that the repair criteria for asymptomatic femoral artery aneurysms should be >35 mm [9]. Furthermore, the presence of an intraluminal thrombus in cases of femoral artery aneurysms is an additional indication for elective repair and may cause ischemic complications [9]. Therefore, surgical decisions must be individualized according to the size of the aneurysm, symptoms, cause of complications, and the patient's general condition. Our surgical indication for elective repair of PFAA is a diameter over 20 mm or symptomatic PFAAs. All patients with PFAAs in this series were treated surgically, and we did not experience any cases of PFAAs that were managed conservatively.

The aim of surgical treatment for PFAAs is to eliminate the risk of complications, including distal ischemia and rupture, and maintain perfusion to the lower extremities. Therefore, surgical repair consists of aneurysmectomy with or without graft replacement [1, 10]. When the superficial femoral artery (SFA) is patent, reconstruction of the PFA is not mandatory; however, in cases with occlusion of the SFA and distal vessels, the PFA serves as an important collateral vessel to the lower extremities and reconstruction is necessary in order to maintain an optimal blood supply. Furthermore, preserving the PFA blood flow may have a positive effect for future limb salvage, as the PFA is less frequently damaged by atherosclerotic changes [3]. Therefore, cases of PFAAs should be treated with both aneurysmectomy and vascular reconstruction, if possible. If the PFAA is located in the distal part of the PFA and/or reconstruction is difficult due to the anatomical location, it may be adequate to excise the aneurysm. Endovascular treatment with stent graft placement is an alternative treatment to ligation, which is a less invasive treatment [11, 12]. In our cases (cases 1 and 5), the aneurysms were located in the distal part of the PFAA and their large size made it difficult to perform reconstruction. Furthermore, both patients had patent SFAs, and we therefore performed aneurysmectomy without graft replacement, which did not lead to ischemic complications.

In conclusion, we herein reported a case series of PFAAs treated surgically with aneurysmectomy with or without graft replacement. Providing an early diagnosis and surgical treatment is necessary to prevent complications, and reconstruction of the PFA is recommended, unless the SFA is patent and performing graft replacement is technically difficult.

## Figures and Tables

**Table 1:** Patients characteristics.

Pt	Gender	Age	PFAA		Clinical symptoms	Diagnostic modality	Other aneurysms	Comorbidity
			Laterality	Size (mm)				
1	M	76	Rt	45 × 40	None	MRI, angiography	AAA	HT, Af, CHF, smoker
2	F	69	Bil	(Rt) 25 × 22 (Lt) 34 × 24	(Rt) None (Lt) Swelling, pain	CT	Bil CFAA	Smoker
3	M	73	Rt	25 × 22	None	CT	TAA, AAA, Bil CIAA	HT, smoker
4	F	65	Rt	26 × 25	None	US, CT	None	Smoker
5	M	70	Lt	86 × 78	Pulsatile mass, pain	CT	None	HT, smoker

^∗^Pt: patient; M: male; F: female; Rt: right; Lt: left; Bil: bilateral; MRI: magnetic resonance imaging; CT: computed tomography; US: ultrasonography; PFAA: profunda femoris artery aneurysm; CFAA: common femoral artery aneurysm; TAA: thoracic aortic aneurysm; AAA: abdominal aortic aneurysm; CIAA: common iliac artery aneurysm; HT: hypertension; Af: atrial fibrillation; CHF: chronic heart failure.

**Table 2:** Surgical procedures and intra- and postoperative findings.

Pt	Surgical procedure	Conduit	Operative time (min)	Intraoperative blood loss (mL)	Pathology
1	Aneurysmectomy	None	149	122	Degenerative

2	(Rt) Aneurysmectomy + revascularization (Lt) Aneurysmectomy + revascularization	(Rt) 8 mm ePTFE + 6 mm ePTFE (Lt) 8 mm ePTFE + 6 mm ePTFE	210	502	(Rt) Degenerative (Lt) Degenerative

3	Aneurysmectomy + revascularization	8 mm Dacron	87	15	Degenerative

4	Aneurysmectomy + revascularization	8 mm ePTFE	130	86	Degenerative

5	Aneurysmectomy	None	81	594	Degenerative

^∗^Pt: patient; ePTFE: expanded polytetrafluoroethylene.

**Table 3:** Postoperative and long-term follow-up results.

Pt	Postoperative morbidity	Postoperative (<30 days) mortality	Follow-up (month)	Limb ischemia	Graft patency
1	None	Alive	8	None	—
2	None	Alive	76	None	Patent
3	Wound infection, relief	Alive	18	None	Patent
4	None	Alive	12	None	Patent
5	None	Alive	35	None	—

^∗^Pt: patient.
